# Fused filament fabrication (FFF): influence of layer height on forces and moments delivered by aligners—an in vitro study

**DOI:** 10.1007/s00784-023-04912-8

**Published:** 2023-02-15

**Authors:** Claudia Spanier, Christian Schwahn, Karl-Friedrich Krey, Anja Ratzmann

**Affiliations:** 1 Department of Orthodontics and Craniofacial Orthopedics, University Medicine Greifswald, 17475 Greifswald, Germany; 2 Department for Prosthodontics, Gerostomatology and Biomaterials, University Medicine Greifswald, Greifswald, Germany

**Keywords:** 3D printing, Force, Fused filament fabrication, In-office aligner, Layer height

## Abstract

**Objectives:**

To investigate the effect of layer height of FFF-printed models on aligner force transmission to a second maxillary premolar during buccal torquing, distalization, extrusion, and rotation using differing foil thicknesses.

**Materials and methods:**

Utilizing OnyxCeph^3^™ Lab (Image Instruments GmbH, Chemnitz, Germany, Release Version 3.2.185), the following movements were programmed for the second premolar: buccal torque (0.1–0.5 mm), distalization (0.1–0.4 mm), extrusion (0.1–0.4 mm), rotation (0.1–0.5 mm), and staging 0.1 mm. Via FFF, 91 maxillary models were printed for each staging at different layer heights (100 µm, 150 µm, 200 µm, 250 µm, 300 µm). Hence, 182 aligners, made of polyethylene terephthalate glycol (PET-G) with two thicknesses (0.5 mm and 0.75 mm), were prepared. The test setup comprised an acrylic maxillary model with the second premolar separated and mounted on a sensor, measuring initial forces and moments exerted by the aligners. A generalized linear model for the gamma distribution was applied, evaluating the significance of the factors layer height, type of movement, aligner thickness, and staging on aligner force transmission.

**Results:**

Foil thickness and staging were found to have a significant influence on forces delivered by aligners, whereas no significance was determined for layer height and type of movement. Nevertheless, at a layer height of 150 µm, the most appropriate force transmission was observed.

**Conclusions:**

Printing aligner models at particularly low layer heights leads to uneconomically high print time without perceptible better force delivery properties, whereas higher layer heights provoke higher unpredictability of forces due to scattering. A *z*-resolution of 150 µm appears ideal for in-office aligner production combining advantages of economic print time and optimal force transmission.

## Introduction 

The Digital Revolution has yielded to the tremendous development of 3D printing reaching back to the early 1980s [1], when the Japanese automobile designer Hideo Kodama first described additive manufacturing [2]. Ever since, the Alaska bald eagle “Beauty”, who lost most of his beak by a poacher’s shot, was able to nourish himself again with the aid of a 3D-printed beak [3]. And 3D-printed prosthetic hands connecting brain impulses via sensors facilitate everyday life of humans, who have lost hands [3]. Moreover, organs, cartilage, and bone 3D bioprinting are well-advanced [1, 4], and NASA is creating 3D-printed prototypes of devices for future space missions [3].

The Digital Revolution is transforming the orthodontic spectrum as well, resulting in a digital workflow including scan, digital treatment planning, and last but not least 3D printing. Using 3D-printing technological creativity has no limits, and orthodontic in-office production of aligner is beginning to flourish.

Despite all the inventive spirit and vast possibilities, the orthodontist has to pay attention to the profitability of his practice. Regarding the different types of 3D printing, namely stereolithography (SLA), digital light processing (DLP), polyjet photopolymer printing (PPP), and fused filament fabrication (FFF), the latter represents the most cost-effective and widest spread technique [1, 5]. Focusing on FFF, layer height is playing a crucial role in modulating manufacturing costs, since increasing layer height leads to exponentially shorter print time, less amount of filament to be used, and thus lowers modeling costs during in-office aligner production [5, 6].

Previous studies have already proven the clinical suitability of FFF in dentistry fields [5, 6]. Moreover, Kamio et al. found no significant geometric decrease in accuracy with increasing layer heights of FFF-printed mandibular jaws varying from 200 to 500 µm. A recent study [6] concluded that there was an optimum range of layer height and found 100 µm being the optimum layer height for lignin-based FFF-printed models, resembling accuracy and precision of DLP models with a layer height of 20 µm. FFF with *z*-resolutions higher than 100 µm were found to further reduce accuracy and precision accompanied by the disadvantages of overall increasing modeling costs.

This study takes one step further ahead, investigating the impact of layer height of FFF-printed models on force delivery of orthodontic aligner to a second upper premolar to optimize in-office aligner production clinically and economically. Moreover, the dependence of planned sequence step, type of movement, and aligner thickness on aligner force transmission was evaluated.

## Materials and methods

### Virtual planning and manufacturing of aligners

**Fig. 1:**
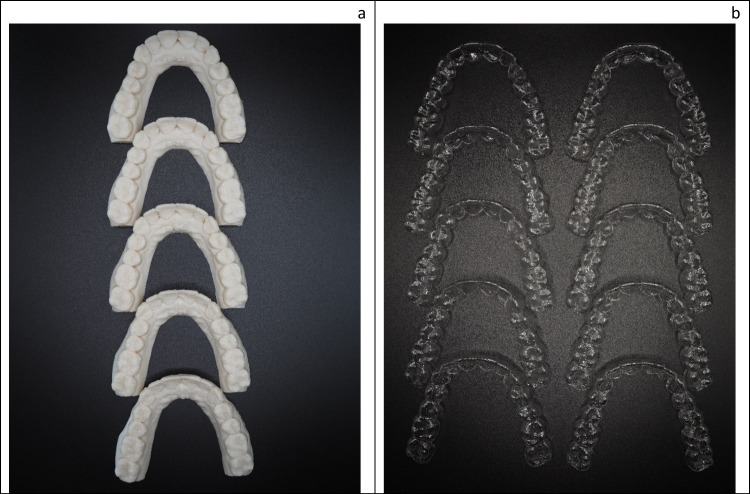
FFF-printed models with planned sequence step of 0.1 mm distalization at different layer heights and their corresponding aligners. **a** FFF models with different layer heights arranged from top to bottom: 100 µm, 150 µm, 200 µm, 250 µm, 300 µm. **b** Corresponding aligners arranged from up to bottom: 100 µm, 150 µm, 200 µm, 250 µm, 300 µm layer height; on the left 0.5 mm aligner series, on the right: 0.75 mm aligner series

With the aid of OnyxCeph^3^™ Lab (Image Instruments GmbH, Chemnitz, Germany), a randomly chosen maxillary arch was digitally modified. Therefore, the following setups for the left second premolar were planned virtually: (a) buccal torque 0.1–0.5 mm in 0.1-mm steps, (b) distalization 0.1–0.4 mm in 0.1-mm steps, (c) extrusion 0.1–0.4 mm in 0.1-mm steps, and (d) rotation 0.1–0.5 mm in 0.1-mm steps. The desired stages of tooth movement, including the initial situation, were exported via STL files and FFF printed (TEVO Tornado, TEVO 3D Electronic Technology, Zhanjiang, China; 0.4-mm nozzle) at different layer heights (100 µm; 150 µm, 200 µm, 250 µm, 300 µm) with a lignin-based polymer (Green-TEC PRO, Extrudr, Lauterach, Austria). Subsequently, 182 orthodontic aligners were manufactured according to the manufacturer’s recommendations via vacuum forming (BIOSTAR®, SCHEU-DENTAL GmbH, Iserlohn, Germany) with two different foil thicknesses (0.5 mm, 0.75 mm) of polyethylene terephthalate glycol (Duran + ®; Scheu Dental GmbH, Iserlohn). All of them were finally cut up to the gingival margin. In the end, 90 active orthodontic aligner and 1 passive calibration aligner were created with a foil thickness of 0.5 mm and a thickness of 0.75 mm each; thus, in total, 182 aligners were formed (Fig. 1).

### Measurement apparatus

**Fig. 2:**
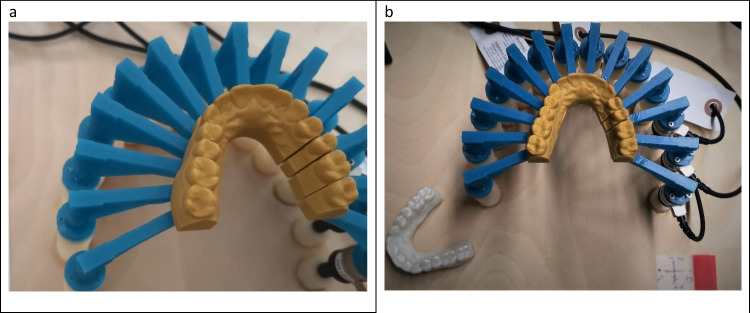
Measurement apparatus M3DOMA. **a** 3D-printed maxillary model with tooth 25 separated. **b** Test apparatus with moment and force sensor 1 connected via column (blue) with tooth 25 — in the lower left, the calibration aligner seated on the corresponding model

To investigate the force transmission to the second premolar via orthodontic aligners, a recently developed 3D-printable force-and-moment-measurement apparatus (M3DOMA) was applied [7] (Fig. 2). The measurement device consisted of two units: an upper part presenting the dental arch to be investigated, with every tooth being attached to an underlying column, and a lower part serving as a moment-and-force sensor (Nano 17, ATI Industrial Automation Inc., Apex, North Carolina, USA). The maxillary arch from the upper part was digitized, converted into a STL file, and DLP printed (SHERAeco-print 30, SHERA Werkstoff-Technologie GmbH & Co. KG, Lemförde, Germany) at a layer height of 50 µm [7]. The upper second premolar was separated mesially and distally to enable tooth movement induced by the seated test aligner. The moment-and-force sensors of the lower unit were interlinked to the tooth to be moved via columns. The sensors were connected to a computer with interposed amplifier. Forces and moments were measured at the tooth’s estimated center of resistance calculated with the aid of the Jacobian matrix [7]. Data analysis was assessed by an individually created program via LabVIEW 2015 15.0f2 (National Instruments Corp., Austin, Texas, USA) [7].

### Test procedure

**Fig. 3:**
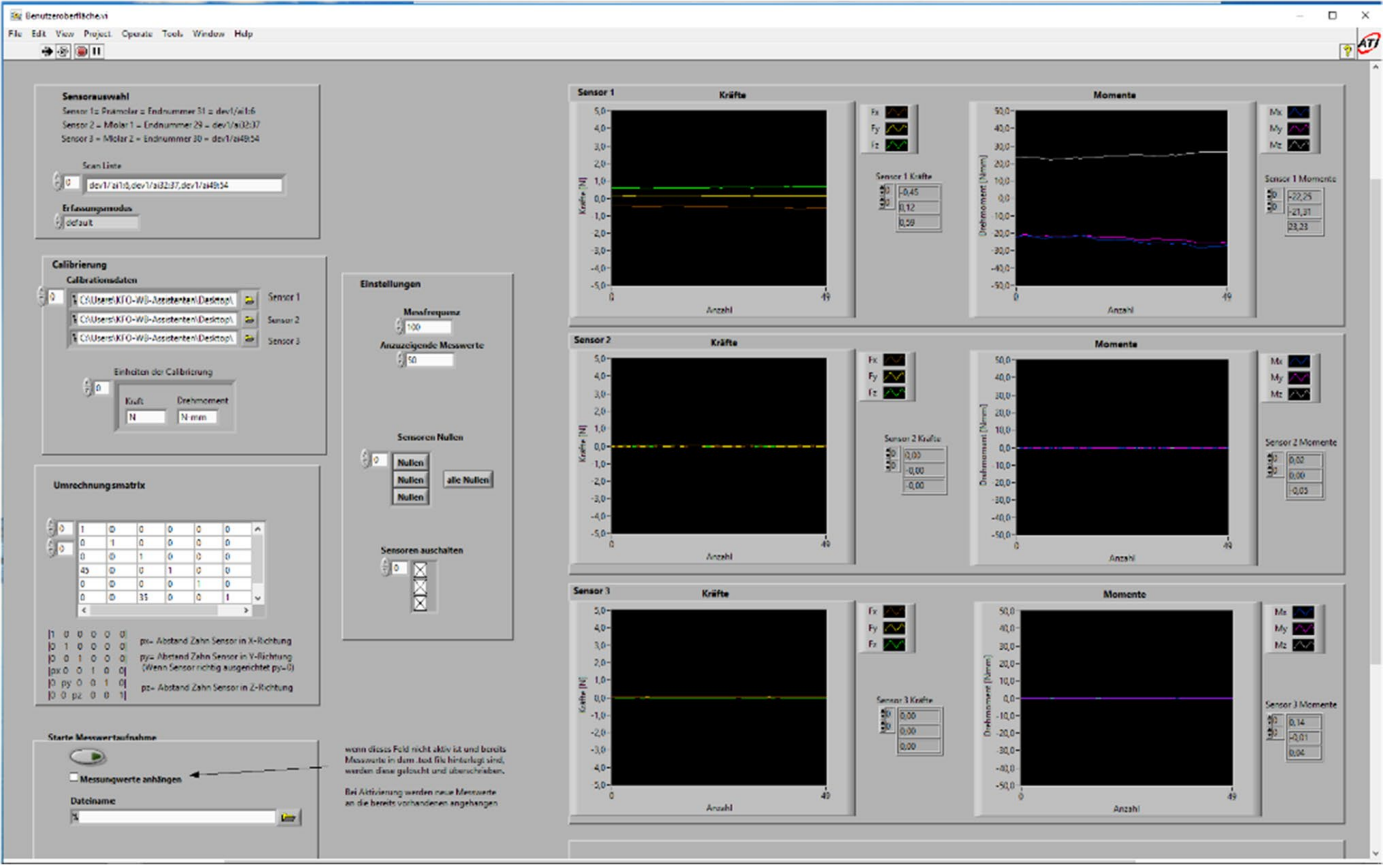
User interface in LabVIEW with measurement output of the second premolar sensor

Via computer program, the experimental setting was determined as follows: measurement frequency, as well as output frequency, was set at 100 Hz. The collected data should be displayed in the physical units N and N mm, respectively (Fig. 3).

For calibration, the passive aligner was seated on the test model at the beginning of each measurement cycle. As soon as the force and moment graphs for the tooth to be investigated settled at 0 N and 0 N mm, respectively, the actual measurement took place. Therefore, the active aligner was seated on the maxillary arch of the measurement apparatus. Subsequently, initial forces and moments transmitted on the second premolar were measured for 10 s. All active aligners were investigated as described.

### Statistical analysis

First, arithmetic means of all measurement data Fx, Fy, Fz, Mx, My, and Mz were calculated with the aid of Excel (Excel 2016, Microsoft Ireland Operations Limited, Dublin, Ireland). Statistical analyses were performed with R and R studio (R Foundation for Statistical Computing, Vienna, Austria). To identifiy influencing parameters on force development, a generalized linear model with gamma distribution was selected and expanded with a post hoc ANOVA; siginificance level was set to 0.05.

## Results

Experiments showed that it is possible to print dental models with the planned layer heights without any problems. As expected, the detail level decreases with increasing layer height. All aligner fitted sufficient on the measurement teeth.

**Fig. 4:**
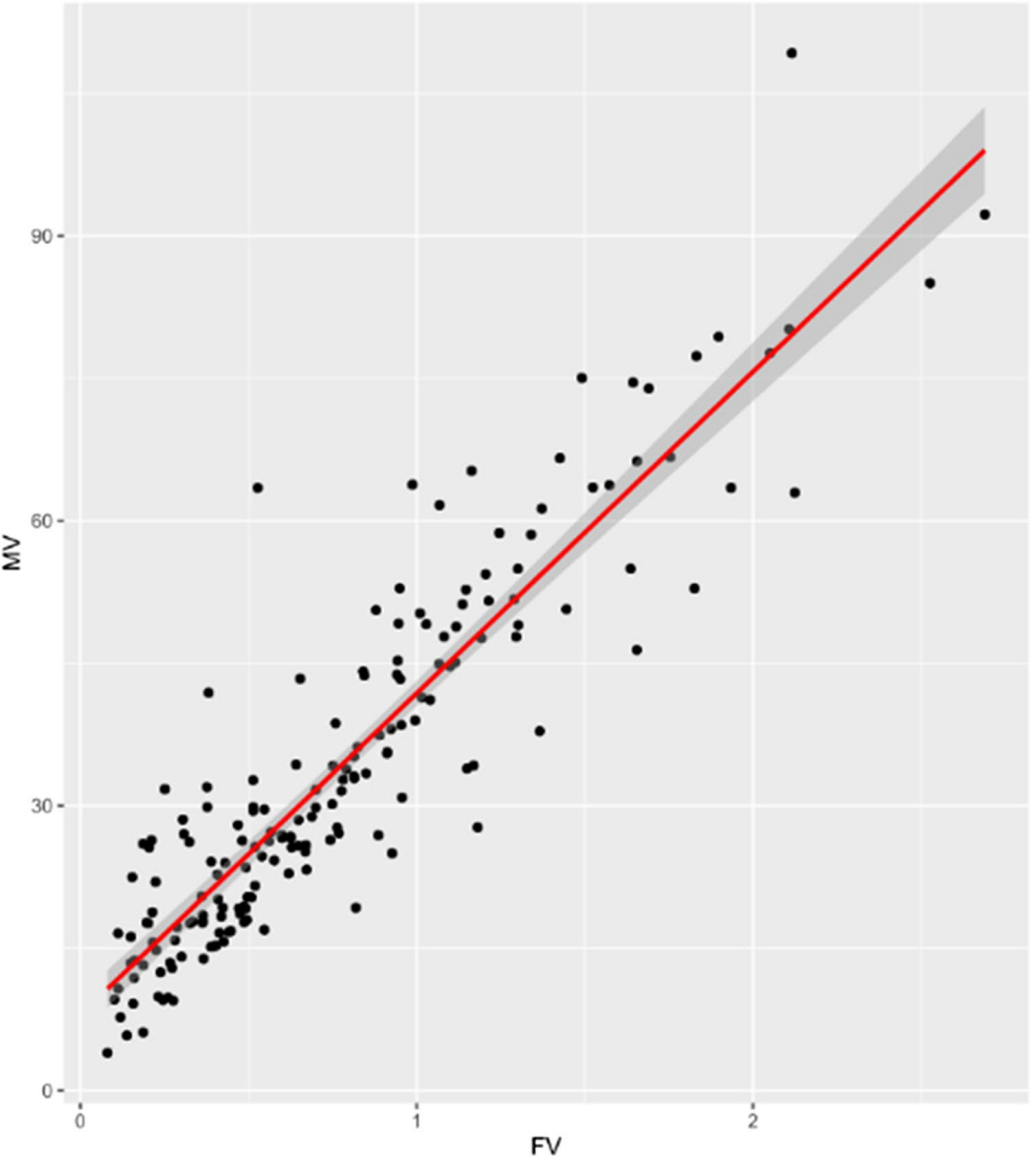
Plot diagram showing a strong correlation of measured moment vectors and force vectors

Comparing the calculated arithmetic means for forces (*F*_x_, *F*_y_, *F*_z_) and moments (*M*_x_, *M*_y_, *M*_z_), direct proportionality was assumed and reinforced by a correlation factor (Pearson) of 0.91 (Fig. 4). For purpose of simplification, the physical parameter moment was neglected. Instead, the resultant force vector, thus the vector sum of the forces Fx, Fy, and Fz, was taken as a reference here.

**Fig. 5:**
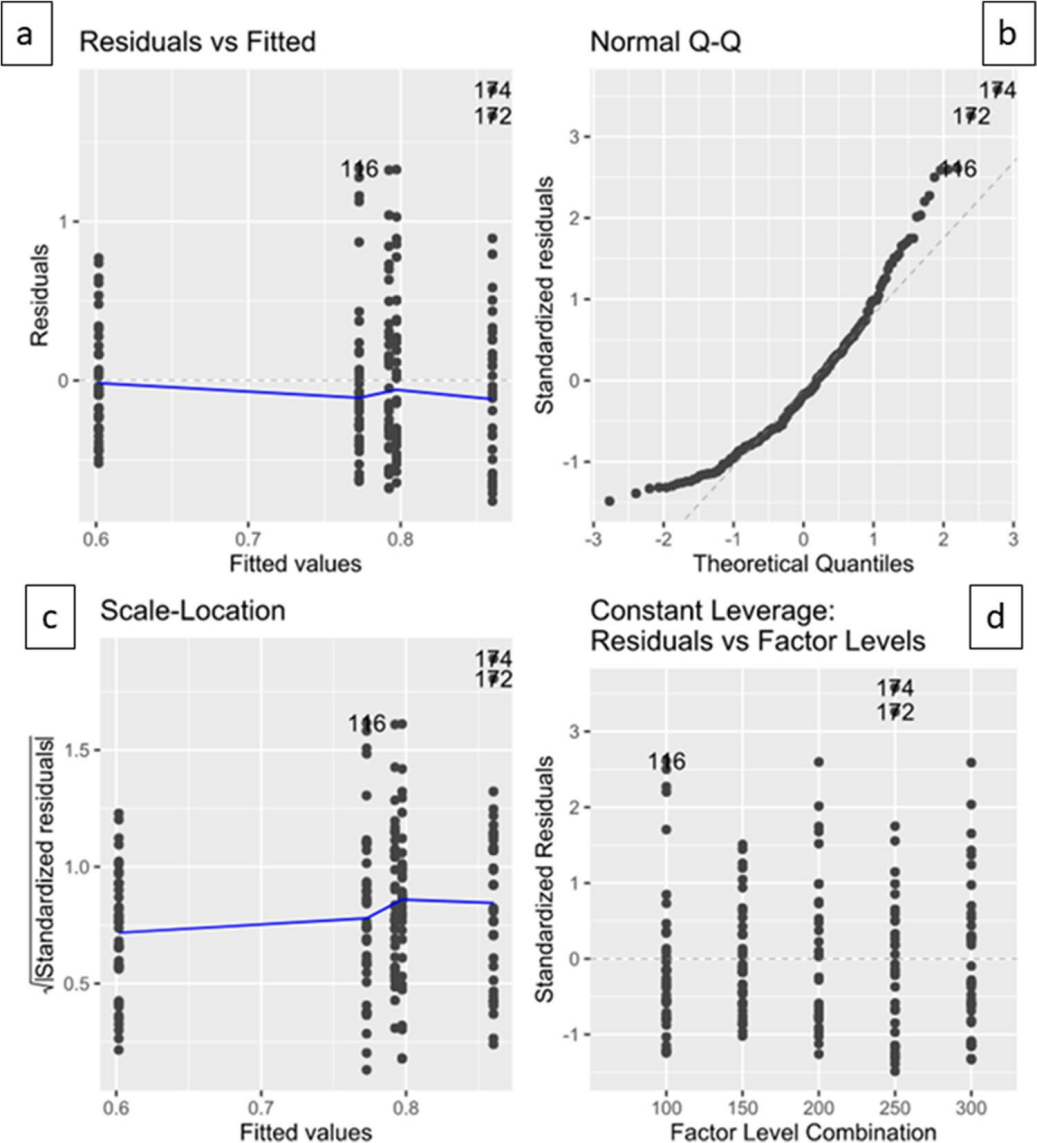
Regression diagnostic plots applied to the measurement data in order to check linear regression assumptions. Scatter plot residuals vs fitted (**a**), normal probability plot (Q-Q-plot) (**b**), scale location plot (**c**), and residuals vs factor levels (**d**)

Analyzing our measuring data to verify normal distribution and linearity, the following diagnostic plots were applied to the assumed statistical model (Fig. 5). The upper left scatterplot residuals versus fitted (Fig. 5a) would support the linear relationship if a horizontal graph without certain patterns was observed. Here, the assumption of linearity seemed doubtful. Normality was checked with the upper right normal probability plot (Q-Q plot) (Fig. 5b); the residual values should therefore follow the straight line — they did not; hence, non-normality of data was estimated. The scale location plot on the lower left (Fig. 5c) was supposed to show equally dispersed points along a horizontal line for constant variances. However, in this case, we saw heteroscedasticity of residuals. The fourth plot (Fig. 5d) depicts residuals versus factor levels to detect possible influential outliers and high leverage points which might affect the regression. In this case, the aligner numbers 116 (100-µm layer height, 0.1-mm distalization, 0.75-mm foil thickness), 172 (250-µm layer height, 0.2-mm rotation, 0.75-mm foil thickness), and 174 (250-µm layer height, 0.4-mm rotation, 0.75-mm foil thickness) were assumed to have possible suspect measurement outcomes.

Since the assumption of normal distribution could be rejected, several statistic models were tested to describe the data set best [8].

Consequently, we found the most appropriate model to be a generalized linear model with the gamma distribution to evaluate the measurement data. Reasons for the assumption were characteristic non-normal distributed, continuous, positive skewed data [8]. Another advantage of the applied model was the ability to compare results by keeping the original scale. The influence of the independent parameters layer height, type of movement, aligner thickness, and staging was examined by the model of likelihood ratio test and the Wald test.

**Fig. 6:**
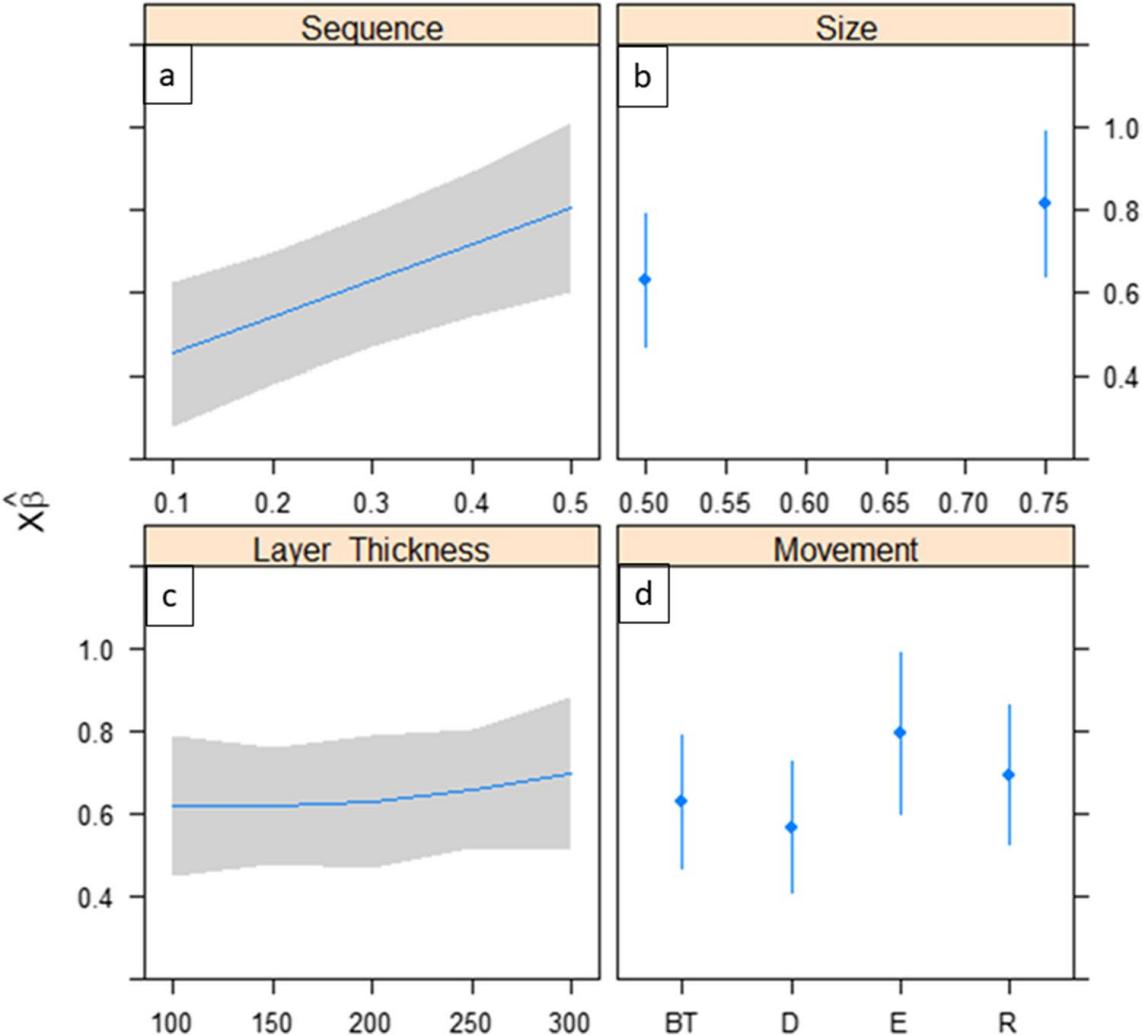
Plot diagrams summarizing the predicted impacts of the investigated factors sequence, foil size, layer height, and type of movement on the resultant forces exerted by aligners on the basis of generalized linear model for the gamma distribution (Xβ). Tooth movements are abbreviated as follows: BT, buccal torque; D, distalization; E, extrusion; R, rotation. Staging step and predicted force vector (**a**), influence of aligner thickness on predicted forces (**b**), relationship between layer height and predicted forces (**c**), and planned movement in relation to predicted force (**d**)

The summary of all investigated factors such as planned sequence, foil size, layer thickness, and the type of movement and their impact on the predicted resulting force vectors according to the implemented generalized linear model with gamma distribution is shown in Fig. 6.

The upper left plot (Fig. 6a) depicts the variable planned sequence (0.1–0.5 mm), that is to say, the staging step and the predicted force vector. The greater the planned dimension of tooth movement, the greater the predicted transmitted forces. Moreover, with larger staging steps than 0.3 mm, scattering of predicted forces increased; hence, the predictability of the force magnitude acting on the tooth decreased.

Looking at the upper right plot (Fig. 6b) representing the influence of aligner foil thickness on the predicted forces, one could presume the following based on the applied statistic model: the higher the foil thickness of the aligner, the higher the sum of forces acting on the tooth. Likewise, there seemed to occur a slightly higher magnitude of force scattering with higher foil thickness.

Regarding the graph (Fig. 6c) displaying the relationship between layer height of the FFF-printed dental cast and the predicted forces, the subsequent assumptions could be drawn: firstly, the pattern of the graph is not as straightforward as the ones in the previous plots. The line of predicted values for force appears to run horizontally and rises slightly from a layer height of 200 µm. Furthermore, the pattern of scattering seems to be smallest at layer heights of 150 µm and 250 µm.

The lower right plot (Fig. 6d) focuses on the parameter type of tooth planned movement about the predicted magnitude of the force acting on the tooth. Apparently, during distalization, the least amount of force was registered followed by buccal torque. On contrary, the highest magnitude of the force exerted by the investigated aligners was measured during extrusion followed by rotation. The scattering follows a similar pattern; it was found to be the least during distalization and the most during extrusion.

**Fig. 7:**
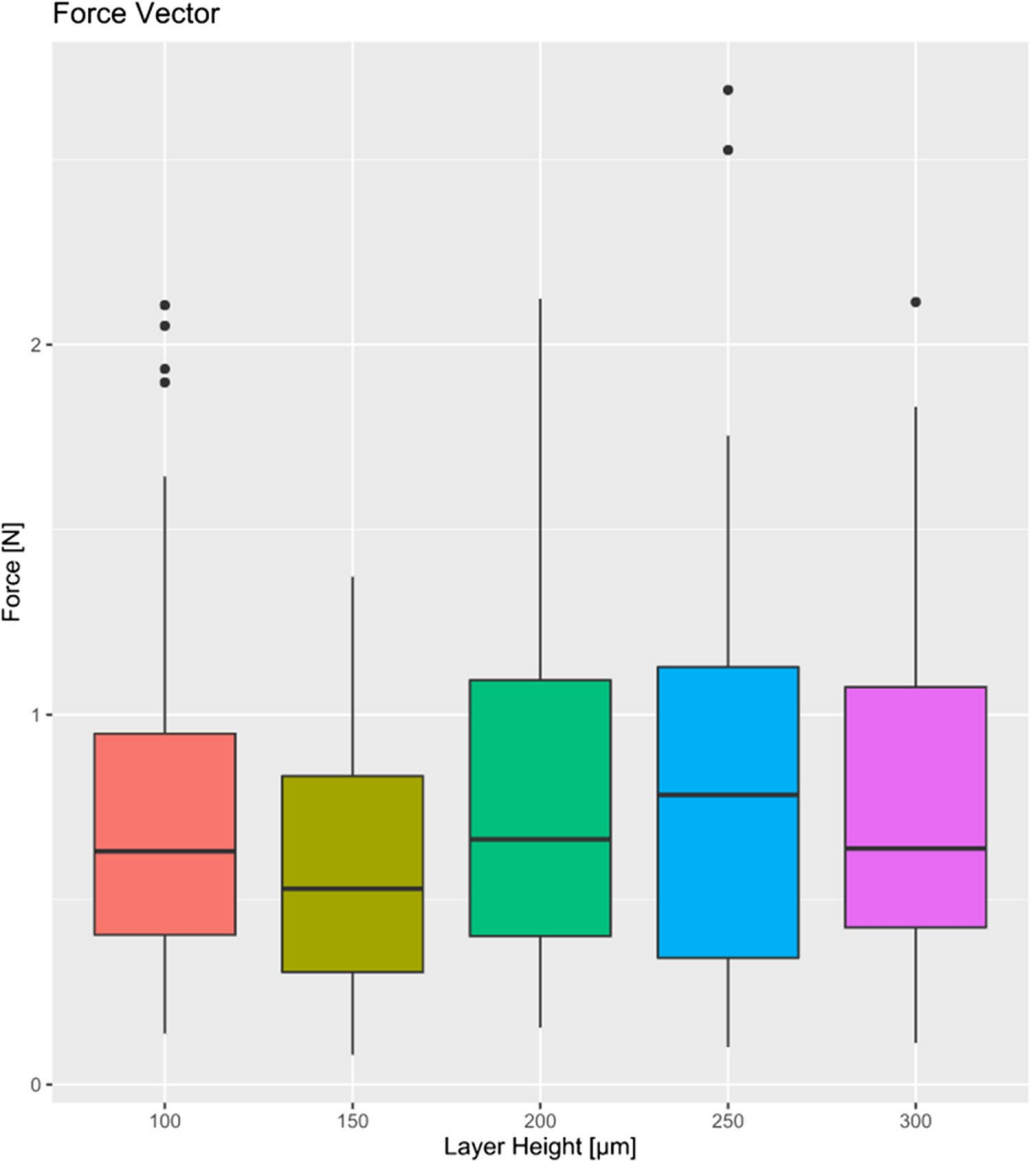
Box plot diagram showing the resultant force vectors transmitted by aligners on the second premolar in dependence of model layer height

In the box plot (Fig. 7), the relation between the force vector and layer height of FFF-printed models is summarized. Similar to the previous observations concerning layer height and its impact on the exerted magnitude of force according to the applied generalized linear model, no clear pattern was recognized. Nonetheless, the force level and its scattering appeared to be the lowest at a layer height of 150 µm. At layer heights smaller or higher than 150 µm, both magnitude of the exerted force and its scattering increased. All in all, aligners originating from FFF-printed models with a layer height of 150 µm seemed to transmit the most appropriate level of force onto the upper second premolar in our in vitro investigation.

**Fig. 8:**
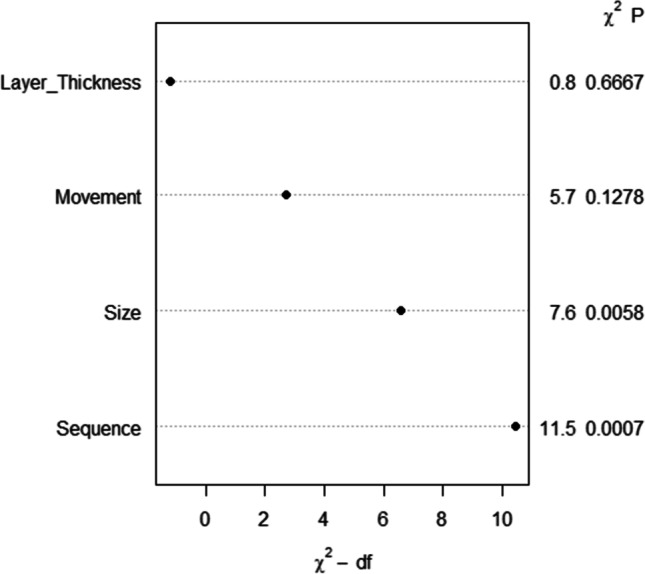
Diagram representing the individual *p*-value of the parameters layer height, type of movement, foil thickness, and sequence step for forces exerted by aligners on the basis of the applied generalized linear model for the gamma distribution, whereas the abbreviations are defined as follows: *χ*^2^ — df. Pearson chi-square divided by its degrees of freedom; *χ*^2^ P, chi-square *p*-value

The following graphical overview (Fig. 8) displays the particular *p*-value for every investigated parameter based on the applied generalized linear model with gamma distribution and the analysis of likelihood ratio test and the Wald statistics. The level of initial force was significantly dependent on the planned sequence step as well as on the size, that is to say, the foil thickness of the aligner. On the contrary, no significant reliance occurred to exist between force level and type of planned tooth movement as well as between force level and layer height of the FFF-printed dental cast.

All in all, force transmission seems to work optimal at a layer height of 150 µm, considering the magnitude and scattering of force; however, the significance could not be proved for this relationship. Rather, statistic significant occurred to be the parameters planned sequence and foil thickness of aligner. Nonsignificant impacts on the magnitude of exerted forces of aligner were observed for the factors planned type of tooth movement as well as layer height of 3D-printed models.

## Discussion

The primary aim of this study was to evaluate the influence of layer height of FFF-printed models on the overall forces exerted by the orthodontic aligner against the backdrop of in-office aligner production. Besides, the dependence of planned sequence, type of movement, and aligner thickness on aligner force transmission was evaluated.

Therefore, forces and moments (Fx, Fy, Fz, Mx, My, Mz) were recorded with a measurement apparatus M3DOMA. According to a previous study [7], the measurement device provided adequate output data given repeatability with a standard deviation less or equal to 0.015 N and reliability with an intraclass correlation for repeated measurements (ICC) ranging from 0.932 to 0.999.

Nonetheless, the statistical analysis detected suspect measurement outcomes — the so-called potential outliers — of the aligner numbers 116, 172, and 174. Taking a closer look at the specific force values, it is striking that especially side effects, such as the intrusive force during rotation among the aligner with the numbers 172 and 174, were characterized by unexpected high values. One reason for those high side effects could lie in error propagation from inaccuracies of FFF-printed models, such as artifacts, to the aligner. Thus, print excess was detected at the originated models of the aligners 172 and 174 at the adjacent teeth of the investigated second premolar and partially transformed into aligner bubbles representing a potential source of error. According to a recent study [6], accuracy of FFF-printed lignin-based models decreased with higher layer heights, which goes with the observation of artifacts on the models that originated from the aligners 172 and 174 each with a layer height of 250 µm. However, the aligner with a foil thickness of 0.5 mm originated from the very same models did not show similar erroneous behavior. Possibly higher foil thickness could lead to higher side effects, and thus, artifacts of models could have more influence on aligner with higher foil thickness. The aligner with the number 116 was originated from a printed model with a layer height of 100 µm. Here, no print artifacts were observed. Another source of error aspect displayed the accuracy of aligner seating during the measuring procedure. To minimize errors on this behalf, only one researcher was involved in the measuring process. Taking all sources of errors into account, it seems most likely that artifacts combined with higher foil thickness yielded outliers. Other possible sources of error for all aligners, inclusively aligner number 116, were irregularities during the vacuum forming [7, 9] as well as possible inconsistencies in the measuring procedure itself.

Looking at the plot summary of the influence of the investigated parameters on the resulting force vector (Fig. 6) and the diagram displaying the *p*-value (Fig. 8), two conclusions can be drawn: firstly, force magnitude can be reduced significantly by the application of thinner aligner foils as already been observed in previous studies [10–15]. Second, tooth overloading and therefore risk of root resorption during aligner therapy could be prevented by planning smaller setup increments likewise concluded in previous studies [11, 16, 17]. Interestingly, in Fig. 6d, movements requiring a smaller tooth loading such as extrusion and rotation had too high observed forces. Even though no significant relationship between the planned type of movement and force transmitted by the aligner was assessed, it might be advisable to plan movements such as extrusion and rotation with smaller increment steps likewise recommended in previous studies [16, 17]. On this behalf, the predictability of extrusion and rotation, previously reported to be the ones with the least efficacy [18–20], might be increased by planning smaller staging steps in addition to attachments.

Evaluating the overall force level in this in vitro study, there are two points to consider. On the one hand side, there is general agreement that the applied amount of orthodontic force should evoke the most efficient tooth movement with the least damage of biological structures such as root resorption for instance [21]. However, in literature, there was no consensus to be found regarding the optimum orthodontic force [22–24], rather than an exact value differing recommended ranges were to be found without a universally valid optimum force level [22, 23]. To give examples, Ricketts proposed an orthodontic force level from 0.45 to 0.75 N for the second upper premolar; another range to be found in recent literature for the very same tooth was 0.40 to 1.2 N [25]. And certainly, more such ranges could be related to [24, 26–29].

Previous studies investigating the impact of orthodontic force on premolar movement even concluded that the amount of achieved tooth movement and the negative side effect of root resorption would not be due to variation of force magnitude but rather due to interindividual structural and metabolic characteristics [27, 30–32]. Nevertheless, other authors pointed out that the lack of impact might occur out of a too narrow applied range of force to distinguish an influence on tooth movement velocity since the optimum force level might be broader than assumed [23]. Others on the contrary found there was a relationship between applied force and root resorption with higher forces leading to a higher risk of orthodontic-induced root resorption [33].

Moreover, the understanding of orthodontic forces implying tooth movement is changing in a way that the previous pressure tension theory might be modulated to a load-dependent reaction of alveolar support structures [34–36], which as well seem to be more complex than assumed so far [34–36]. In other words, not the force magnitude itself to the affected root surface and the distributed stress and tension areas of the periodontal ligament (PDL) but rather the force magnitude to the individual alveolar support structure characteristics and their micromorphology seems to be of crucial importance for the desired tooth movement and therefore patient specific [34].

On the other hand, it seems expedient to compare the measuring results in this study to the recommended force values from the literature. Previous studies concluded that considerable tooth overloading was measured when investigating aligner force behavior in vitro [10, 11, 37], referring to the force level recommended by Proffit [26]. Taking a closer look at our results, one could state that a large portion of measured values at a model layer height of 150 µm (Fig. 7) promises an adequate tooth loading, as the boxplot roughly lies within the optimum force range of 0.4–1.2 N for a second premolar according to the literature [25]. Even when comparing with Proffit’s similar recommendations, we come to the same conclusion. Nonetheless, the upper whisker of the 150 µm boxplot contains measured forces exceeding the recommended tooth loading. Still, against the background of changing principles concerning applied orthodontic forces and tooth movement, further research is required in order to make a clear statement here.

Even though exceedingly high initial forces were observed partly in this as well as in other in vitro studies [10, 11, 37], in clinic routine, aligner therapy seems to yield to similar [38–40] or even less [41, 42] risk of orthodontic-induced root resorption when compared to treatment with fixed appliances, indicating adequate loading by the aligner. Why would that be? One enlightening reason lies in the characteristic force delivery of aligners, due to their viscoelastic properties: previous studies observed a rapid, exponentially appearing force decay during the first hours or first day after loading [43–46], respectively, with further, but more plateau-appearing reduction during the following days [43, 46]. Thus, a recent study by Elkholy et al. [46] investigating force decay of PET-G aligner found a stress relaxation ranging from roughly 80–60% on the first day of loading and overall stress relaxation of 70–95% after 7 days depending on aligner thickness and load regimen. Second, the elasticity of PDL reduces the risk of overloading, buffering the initial force by dampening effect and reduction of discrepancy of the actual tooth position and programmed tooth by physiological dental mobility. Therefore, partly exceeding forces measured in this in vitro study are likely to be attenuated by the dampening effect of PDL and the rapid force diminish of the aligner [46].

Here, we come to the limitations of this in vitro study, which were similar to previous in vitro studies investigating aligner mechanics [11, 13, 37, 47–49]. Due to the experimental setting with the rigid connection between the model tooth and measurement sensor, the characteristic behavior of the PDL with its dampening effect could not be taken into account. Moreover, the influence of the oral environment with its saliva, supposed to be responsible for hygrothermal aging of aligners [44], as well as the impact of the physiological act of swallowing and grinding, was not examined. In addition, only one tooth was moved experimentally, whereas in clinical practice adjacent teeth get movement impulses too. Therefore, future studies are necessary, considering the abovementioned-specific clinical influences.

Apart from the clinical shortcomings, the present study was primarily designed to investigate the influence of layer height of FFF models on aligner force transmission during in-office production. The economically interesting factor layer height does not seem to play a significant role during force delivery of orthodontic aligners. However, the least scattering of measured force values was observed at a *z*-resolution of 150 µm (Fig. 7) limiting the risk of unpredictable side effects. Furthermore, the force level appeared to be optimal at a layer height of 150 µm, reducing exceedingly high initial aligner forces and therefore preventing undesired side effects such as root resorption. A *z*-resolution of FFF-printed dental casts lower than 150 µm would lead to an increment of unpredictable forces exerted by aligners, whereas a *z*-resolution higher than 150 µm would result in exponentially increasing print time as well as higher material consumption and therefore higher manufacturing costs. All in all, FFF-printed models with a layer height of 150 µm were found to realize the requirements for in-office aligner production best, combining the advantages of clinical and economic efficacy. For further investigation of FFF optimum layer height concerning aligner production, future studies additionally implementing auxiliaries such as attachments or power ridges would be desirable.

## Conclusions

Taking the results of this study into account, there seems to be no reasonable advantage in printing FFF models with particularly low layer heights for in-office aligner manufacturing not leading to better force transformation and facing economic inefficiency with exponentially higher printing time.

However, higher layer heights provoke higher scattering of resultant force leading to a higher unpredictability of aligner force transmission.

An ideal compromise represents a layer height of 150 µm. Here, the force level is optimal, and its scattering is minimal resulting in less undesired, unpredictable forces.

Considering force delivery of orthodontic aligners, the parameters planned sequence step and foil thickness appear to be statistically significant.

In clinical application, FFF-printed models should be checked for artifacts before aligner production.

## Data Availability

Data is available via the corresponding author.
